# Clinical risk, sociodemographic factors, and SARS-CoV-2 infection over time in Ontario, Canada

**DOI:** 10.1038/s41598-022-13598-z

**Published:** 2022-06-24

**Authors:** Jacob A. Udell, Bahar Behrouzi, Atul Sivaswamy, Anna Chu, Laura E. Ferreira-Legere, Jiming Fang, Shaun G. Goodman, Justin A. Ezekowitz, Kevin R. Bainey, Sean van Diepen, Padma Kaul, Finlay A. McAlister, Isaac I. Bogoch, Cynthia A. Jackevicius, Husam Abdel-Qadir, Harindra C. Wijeysundera, Dennis T. Ko, Peter C. Austin, Douglas S. Lee

**Affiliations:** 1 ICES, Toronto, Canada; 2 Cardiovascular Division, Department of Medicine, Women’s College Hospital, Toronto, Canada; 3 Peter Munk Cardiac Centre, University Health Network, Toronto, Canada; 4 Institute of Health Policy, Management, and Evaluation, University of Toronto, Toronto, Canada; 5 Department of Medicine, Temerty Faculty of Medicine, University of Toronto, Toronto, Canada; 6 Division of Cardiology, St. Michael’s Hospital, Toronto, Canada; 7 Canadian VIGOUR Centre, University of Alberta, Edmonton, Canada; 8 Department of Medicine, Faculty of Medicine and Dentistry, University of Alberta, Edmonton, Canada; 9 Department of Critical Care Medicine and Division of Cardiology, Department of Medicine, University of Alberta, Edmonton, Canada; 10 Divisions of General Internal Medicine and Infectious Diseases, University Health Network, Toronto, Canada; 11 Western University of Health Sciences, Pomona, CA USA; 12 Schulich Heart Centre, Sunnybrook Health Sciences Centre, Toronto, Canada

**Keywords:** Risk factors, Epidemiology, Epidemiology, Viral infection

## Abstract

We aimed to determine whether early public health interventions in 2020 mitigated the association of sociodemographic and clinical risk factors with severe acute respiratory syndrome coronavirus 2 (SARS-CoV-2) infection. We conducted a population-based cohort study of all adults in Ontario, Canada who underwent testing for SARS-CoV-2 through December 31, 2020. The outcome was laboratory-confirmed SARS-CoV-2 infection, determined by reverse transcription polymerase chain reaction testing. Adjusted odds ratios (ORs) were determined for sociodemographic and clinical risk factors before and after the first-wave peak of the pandemic to assess for changes in effect sizes. Among 3,167,753 community-dwelling individuals, 142,814 (4.5%) tested positive. The association between age and SARS-CoV-2 infection risk varied over time (*P*-interaction < 0.0001). Prior to the first-wave peak, SARS-CoV-2 infection increased with age whereas this association reversed thereafter. Risk factors that persisted included male sex, residing in lower income neighborhoods, residing in more racially/ethnically diverse communities, immigration to Canada, hypertension, and diabetes. While there was a reduction in infection rates after mid-April 2020, there was less impact in regions with higher racial/ethnic diversity. Immediately following the initial peak, individuals living in the most racially/ethnically diverse communities with 2, 3, or ≥ 4 risk factors had ORs of 1.89, 3.07, and 4.73-fold higher for SARS-CoV-2 infection compared to lower risk individuals in their community (all *P* < 0.0001). In the latter half of 2020, this disparity persisted with corresponding ORs of 1.66, 2.48, and 3.70-fold higher, respectively. In the least racially/ethnically diverse communities, there was little/no gradient in infection rates across risk strata. Further efforts are necessary to reduce the risk of SARS-CoV-2 infection among the highest risk individuals residing in the most racially/ethnically diverse communities.

## Introduction

In late 2019, the novel coronavirus disease 2019 (COVID-19) emerged as a worldwide pandemic threat^1^. By early 2020, virus transmission began across North America. Transmission and cases of severe acute respiratory syndrome coronavirus 2 (SARS-CoV-2) infection developed earlier and in more rapid succession in certain cities with densely populated and marginalized populations^2,3^. Other countries had some lead time to prepare for SARS-CoV-2 infections, including Canada, which has an overall smaller and more geographically distributed population size and lower scale of international travel that could influence the regional epidemiology of COVID-19.

Ontario is the most populous province in Canada with diversity in age, socioeconomic status, and race/ethnicity across the province’s counties, townships, and municipalities^4^. While the presence of certain clinical characteristics, particularly a history of cardiovascular and renal disease or obesity, may increase the likelihood of SARS-CoV-2 infection^5–13^, other data show sociodemographic characteristics, including race and socioeconomic status, may be stronger drivers of infection risk as seen with other communicable diseases^14–25^. However, earlier data may be biased if cases were selected only among individuals presenting to hospital, with limited or no controls. Furthermore, estimates of risk may be biased by the propensity for or against exposure and testing over time. Therefore, we sought to evaluate the association of sociodemographic and clinical risk factors with the likelihood of laboratory-confirmed SARS-CoV-2 infection before and after the peak of the first wave and until the end of 2020 in Ontario, by analyzing linked population-based health databases among individuals tested for SARS-CoV-2. Given that the time period surrounding the peak of the first wave coincided with the introduction of a broad suite of public health measures aimed at mitigating the spread of the pandemic, we further describe whether instituting these public health interventions in March/April of 2020 (Supplemental Fig. 1) were effective at mitigating both sociodemographic and clinical risk factors.

## Methods

### Study design and population

Ontario has a publicly funded health care system with universal access to care without user fees at the point of service. We assembled a population-based retrospective cohort of all Ontarians aged 18 years and older who were eligible for the province’s universal Ontario Health Insurance Plan (OHIP), alive as of January 1, 2020, and who underwent testing for SARS-CoV-2 up to December 31, 2020 (Supplemental Fig. 2). This cohort was created at ICES, a non-profit research institute whose legal status under Ontario’s health information privacy law allows it to collect and analyze health care and demographic data, without consent, for health system evaluation and improvement. The cohort was created through linkage of multiple provincial and federal health care related databases (e.g., hospital discharge abstracts, physician claims, chronic disease registries, health survey, laboratory, and drug dispensing data), as well as the Immigration, Refugees and Citizenship Canada (IRCC) Permanent Resident database^26^. These datasets were linked using unique, encoded identifiers and analyzed at ICES as previously described and validated^27^. We required individuals to be eligible for health insurance one year prior to the index date of study as determined from the Ontario Registered Persons Database (RPDB), which includes basic demographic information about anyone who has ever had an Ontario health insurance number. Individuals who were not residents of Ontario on the index date were excluded. Since baseline health status and congregate living arrangements of long-term care residents differ substantially from community-dwelling individuals, they were also excluded from these analyses. The index date for study inclusion was the date of a first SARS-CoV-2 test, either as recorded in the Ontario Laboratories Information System (OLIS; which consolidated the majority [≥ 88%] of results of COVID-19 testing during the first wave in Ontario), and/or the Case and Contact Management System (CCM).

### Exposure variables

Testing date was divided into calendar weeks. We obtained data on age, sex, and community-dwelling characteristics from the RPDB. Communities were categorized by regional public health units (PHUs; also referred to as “regions” throughout), geographic location, and size according to Statistics Canada’s Census data^28^. Communities with less than 10,000 residents were classified as rural. Median neighborhood income was categorized by quintile according to national Census data. Individuals who immigrated to Ontario as their first place of landing in Canada between 1985 and 2017 were identified via the IRCC Permanent Resident database.

Clinical comorbidities were identified using previously validated case definition algorithms for Canadian administrative databases based on hospitalization and emergency department records from the Canadian Institute for Health Information Discharge Abstract Database (CIHI DAD) and National Ambulatory Care Reporting System (NACRS), respectively using International Classification of Diseases, Tenth Revision, Canada (ICD-10-CA) coding, hospital and physician procedure coding, and chronic disease diagnoses from the OHIP database (Supplemental Table 1). In addition to the number of hospitalization or emergency department episodes in the prior year, we also included the following characteristics within the previous 5 years: history of coronary artery disease (CAD; defined as a prior myocardial infarction, percutaneous or surgical coronary revascularization); hospitalization for heart failure (HF) or stroke, history of liver disease, chronic lung disease (including pneumonia, tuberculosis, asthma or chronic obstructive pulmonary disease), organ transplantation, atrial fibrillation, chronic kidney disease (CKD) or malignant cancer. Any prior history and duration of hypertension, any history of diabetes or human immunodeficiency virus (HIV) was also assessed. Frailty was defined using the Johns Hopkins Adjusted Clinical Groups (ACG®) Version 10 frailty indicator^29,30^. Sex-specific and, when available, age-standardized regional rates of smoking, obesity, and racial/ethnic diversity were calculated at the public health unit level using national census and survey data available from Public Health Ontario due to a lack of individual-level administrative data on income and race in Ontario and Canada more generally^4,31–34^. Racial/ethnic diversity was defined as the estimated regional visible minority proportion of individuals who self-identified as Black, South Asian, Chinese, Filipino, Latin American, Arab, Southeast Asian, West Asian, Korean, and Japanese according to national Census data^35^. The receipt of influenza vaccination within the past year was determined from the OHIP and ODB databases among eligible individuals.

### Outcomes

Our outcome of interest was laboratory-confirmed SARS-CoV-2 infection, via the proxy of testing positive for SARS-CoV-2 by reverse transcription polymerase chain reaction (RT-PCR). As individuals could undergo multiple tests over the study period, if any test was positive, they were classified as having the outcome. Otherwise, individuals testing negative for SARS-CoV-2 in all tests comprised the uninfected group.

### Statistical analysis

Sociodemographic and clinical characteristics between individuals with and without laboratory-confirmed SARS-CoV-2 infection were compared using chi-square tests for categorical variables and one-way analysis of variance for continuous variables.

To evaluate the association between baseline characteristics and risk of SARS-CoV-2 infection, we fit multivariable logistic regression models to determine the odds ratio (OR) of testing positive. We regressed the outcome (testing positive vs. testing negative) on sociodemographic and clinical characteristics, incorporating PHU-specific random effects to account for clustering of individuals within communities. In these multivariable analyses, we adjusted for calendar week of testing, sociodemographic factors (age, sex, rural/urban residence, immigrant status, neighborhood income quintile, and regional racial/ethnicity diversity rate), and the aforementioned clinical risk factors. An interaction term was introduced to test for heterogeneity in the association of age with the likelihood of SARS-CoV-2 infection over testing week. Given significant heterogeneity was detected, we stratified reporting of results into three time periods, one prior to the peak of the first wave of the pandemic, one immediately following the peak, and the latter half of 2020.

Sociodemographic and clinical risk factors that were independently associated with laboratory-confirmed SARS-CoV-2 infection were then used to calculate an integer risk score for each individual by summing the number of risk factors the individual had. Separately for the periods prior to and following the peak of the first wave of the pandemic, we then calculated absolute infection rates and the odds ratio of SARS-CoV-2 infection in Ontario stratified by quartiles of regional rates of racial/ethnic diversity (Ontario median 4.0%, interquartile range, 2.5–17.6%; Supplemental Fig. 3). Associations of the integer scores with SARS-CoV-2 infection were assessed using logistic regression models. As few individuals had no risk factors following the peak of the pandemic, we combined the presence of 0 to 1 risk factors as the lowest risk group for the post-peak period. Trend in infection risk with increasing integer score was assessed using the Cochrane-Armitage trend test. All *P* values were 2-sided with *P* < 0.05 considered significant. The study followed the Strengthening the Reporting of Observational Studies in Epidemiology (STROBE) reporting guideline. All data were analyzed at ICES using SAS version 9.4 (SAS Institute, Cary, NC).

### Ethical statement

ICES is a prescribed entity under section 45 of Ontario’s Personal Health Information Protection Act. Section 45 authorizes ICES to collect personal health information, without consent, for the purpose of analysis or compiling statistical information with respect to the management of, evaluation or monitoring of, the allocation of resources to, or planning for all or part of the health system. Projects conducted under section 45, by definition, do not require review by a research ethics board (for use of anonymized data). The data access and analysis for this study was conducted under section 45 and all relevant protocols/materials were approved by ICES’ Privacy and Compliance Office. All methods were carried out in accordance with locally relevant guidelines and regulations.

## Results

**Figure 1:**
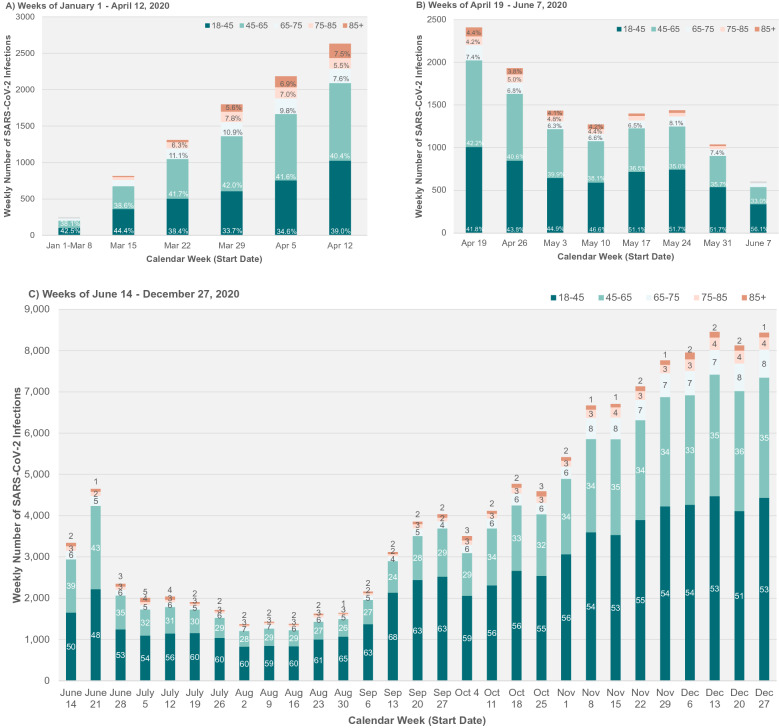
Weekly number and rate of community-dwelling individuals with SARS-CoV-2 infection stratified by age group during 2020, prior to (**A** Weeks of January 1–April 12) and following (**B** Weeks of April 19–June 7 and **C** Weeks of June 14–December 27) the peak of the first wave of the pandemic in Ontario, Canada. Time represented by the start of the calendar week with the weeks of January 1 through March 8 consolidated given low initial infection counts. Age groups: 18–45 years (dark green); 45–65 years (light green); 65–75 years (light blue); 75–85 years (pink); 85 years and older (orange). The proportion of weekly counts attributable to an age group is represented in each column with the percentage indicated on each section. The peak of the first wave of the pandemic regarding community-dwelling infections, using the proxy of test positivity in Ontario, correlated with the week of April 12, 2020. Ages 75 and up are combined to suppress small cells for weeks Jan 1 – Mar 8 and June 7, per ICES' reidentification risk assessment procedures.

A description of the number of study participants and reasons for inclusion and exclusion are summarized in Supplemental Fig. 2. Overall, 3,167,753 eligible community-dwelling patients underwent RT-PCR testing for SARS-CoV-2 infection between January 1 and December 31, 2020, of which 142,814 (4.5%) had confirmed test positivity (Table 1 and Supplemental Table 2). The weekly number and rate of community-dwelling individuals first testing positive for SARS-CoV-2 by age group is presented in Fig. 1. The weekly number of community-dwelling individuals tested for SARS-CoV-2 and share of tests that were positive, stratified by age group, is presented in Supplemental Fig. 4. During the first wave, the peak number and proportion of individuals with laboratory-confirmed SARS-CoV-2 infection occurred during the week starting April 12, 2020, three weeks following a province-wide lockdown, which reflects the standard latency period for measuring impact of public health measures on infection rates (Supplemental Fig. 1)^36^.

**Table 1:** Baseline characteristics of community-dwelling patients with and without SARS-CoV-2 infection during the first wave of the pandemic in Ontario, Canada.

Characteristic		Negative test (n = 557,739)	Positive test (n = 20,524)	SD	*P* value
Week of testing (start date)	0–10 (January 1–March 8)	8477 (1.5%)	252 (1.2%)	0.03	< 0.001
	11 (March 15)	16,069 (2.9%)	816 (4.0%)	0.06	
	12 (March 22)	15,203 (2.7%)	1311 (6.4%)	0.18	
	13 (March 29)	15,921 (2.9%)	1798 (8.8%)	0.25	
	14 (April 5)	19,749 (3.5%)	2184 (10.6%)	0.28	
	15 (April 12)	34,058 (6.1%)	2632 (12.8%)	0.23	
	16 (April 19)	42,864 (7.7%)	2410 (11.7%)	0.14	
	17 (April 26)	52,234 (9.4%)	1932 (9.4%)	0	
	18 (May 3)	55,546 (10.0%)	1435 (7.0%)	0.11	
	19 (May 10)	45,758 (8.2%)	1272 (6.2%)	0.08	
	20 (May 17)	33,468 (6.0%)	1400 (6.8%)	0.03	
	21 (May 24)	71,835 (12.9%)	1439 (7.0%)	0.20	
	22 (May 31)	78,056 (14.0%)	1037 (5.1%)	0.31	
	23 (June 7)	68,501 (12.3%)	606 (3.0%)	0.36	
**Sociodemographic factors**					
Age, years		51 (35–64)	49 (34–61)	0.09	< 0.001
Age group, years	18–45	230,307 (41.3%)	8789 (42.8%)	0.03	< 0.001
	45–65	195,925 (35.1%)	8130 (39.6%)	0.09	
	65–75	59,943 (10.7%)	1683 (8.2%)	0.09	
	75–85	40,276 (7.2%)	1053 (5.1%)	0.09	
	≥ 85	31,288 (5.6%)	869 (4.2%)	0.06	
Sex	Female	344,040 (61.7%)	11,186 (54.5%)	0.15	< 0.001
	Male	213,699 (38.3%)	9338 (45.5%)	0.15	
Income quintile	1 (lowest)	122,004 (21.9%)	5827 (28.4%)	0.15	< 0.001
	2	115,955 (20.8%)	4343 (21.2%)	0.01	
	3	111,368 (20.0%)	4168 (20.3%)	0.01	
	4	106,160 (19.0%)	3361 (16.4%)	0.07	
	5 (highest)	102,252 (18.3%)	2825 (13.8%)	0.12	
Community size	< 10,000	63,441 (11.4%)	743 (3.6%)	0.30	< 0.001
	10,000–100,000	53,810 (9.6%)	832 (4.1%)	0.22	
	100,000–500,000	134,283 (24.1%)	2543 (12.4%)	0.31	
	500,000–1.5 million	87,383 (15.7%)	2427 (11.8%)	0.11	
	≥ 1.5 million	218,822 (39.2%)	13,979 (68.1%)	0.61	
Canadian immigrant		94,187 (16.9%)	7836 (38.2%)	0.49	< 0.001
Regional smoking rate, %		12 (9–17)	12 (9–13)	0.41	< 0.001
Regional obesity rate, %		21 (15–24)	16 (15–22)	0.47	< 0.001
Regional racial/ethnic diversity rate, %		19 (7–51)	51 (25–52)	0.65	< 0.001
**Clinical risk factors**					
Hypertension		174,916 (31.4%)	6317 (30.8%)	0.01	0.077
Hypertension duration, years		13 (7–20)	12 (6–19)	0.14	< .001
Diabetes		66,994 (12.0%)	2916 (14.2%)	0.07	< .001
Coronary artery disease		24,352 (4.4%)	619 (3.0%)	0.07	< .001
Heart failure		11,921 (2.1%)	308 (1.5%)	0.05	< .001
Stroke		4576 (0.8%)	158 (0.8%)	0.01	0.429
Atrial fibrillation		24,555 (4.4%)	631 (3.1%)	0.07	< .001
Chronic kidney disease		27,673 (5.0%)	854 (4.2%)	0.04	< .001
HIV		1630 (0.3%)	56 (0.3%)	0	0.613
Organ transplantation		1390 (0.2%)	31 (0.2%)	0.02	0.005
Cancer		19,324 (3.5%)	401 (2.0%)	0.09	< .001
Liver disease		3502 (0.6%)	78 (0.4%)	0.04	< .001
Lung disease		129,702 (23.3%)	3742 (18.2%)	0.12	< .001
Hospitalization or ED visits in 2019	0	261,024 (46.8%)	11,254 (54.8%)	0.16	< .001
	1–2	186,021 (33.4%)	6522 (31.8%)	0.03	
	3 +	110,694 (19.8%)	2748 (13.4%)	0.17	
Frailty		32,041 (5.7%)	995 (4.8%)	0.04	< .001
Influenza vaccination in 2019		163,565 (29.3%)	4792 (23.3%)	0.14	< .001

Continuous variables are reported as median (interquartile range).

In the early stages of modelling, there was significant heterogeneity between age and week of testing (*P*-interaction < 0.0001), hence results are presented stratified prior to and following the peak of the first wave of the pandemic until the end of 2020 (Supplementary Table 2).

*ED* emergency department, *HIV* human immunodeficiency virus, *SD* standardized difference.

Baseline sociodemographic and clinical characteristics among community-dwelling individuals with and without SARS-CoV-2 infection during the first wave (i.e., the first half of 2020) are presented in Table 1. Compared with individuals without SARS-CoV-2 infection, community-dwelling individuals with SARS-CoV-2 infection were overall younger, more frequently male, immigrants, and residing in racially/ethnically diverse, large, urban, low-income communities. This trend continued until the end of 2020 (Supplementary Table 2). Furthermore, individuals with SARS-CoV-2 infection had higher rates of diabetes initially, but otherwise had lower rates of most clinical comorbidities and lower rates of recent hospitalization or ED visits, which remained consistent until the end of 2020.

Independent predictors of laboratory-confirmed SARS-CoV-2 infection among community-dwelling individuals tested are presented in Table 2. Results are stratified into the period prior to and immediately following the peak of the first wave of the pandemic in the province, as well as the remainder of 2020, given the detection of significant heterogeneity in the risk of SARS-CoV-2 infection by age across time (*P*-interaction < 0.0001). During the period leading up to the peak of the first wave of the pandemic, the likelihood of SARS-CoV-2 infection progressively increased across age groups (age 45–65, OR 1.30, 95% CI 1.24–1.37; age 65–75, OR 1.38, 95% CI 1.26–1.51; age 75–85, OR 1.46, 95% CI 1.30–1.63; age 85 and older, OR 1.60, 95% CI 1.41–1.81) compared with the youngest individuals aged 18–45 years. Other independent risk factors for SARS-CoV-2 infection included male sex (OR 1.45, 95% CI 1.31–1.60), residing in the lowest quintile of neighborhood income (OR 1.09, 95% CI 1.01–1.17), residing in more racially/ethnically diverse communities (OR per 1% increase in regional racial/ethnic diversity 1.02, 95% CI 1.01–1.03), immigration to Canada (OR 1.53, 95% CI 1.45–1.61), frailty (OR 1.31, 95% CI 1.01–1.25), hypertension (OR 1.10, 95% CI 1.04–1.17), and diabetes (OR 1.12, 95% CI 1.05–1.20).

**Table 2:** Predictors of SARS-CoV-2 infection prior to (weeks of January 1 to April 12, 2020) and following (weeks of April 19 to June 7, 2020; weeks of June 14 to December 27, 2020) the peak of the first wave of the pandemic among community-dwelling individuals in Ontario, Canada.

Characteristic		OR (95% CI)	*P* value
**Weeks of January 1–April 12, 2020**			
Week of testing (start date)	0–10 (January 1–March 8)	Referent	< 0.0001
	11 (March 15)	1.95 (1.69–2.26)	
	12 (March 22)	3.63 (3.16–4.18)	
	13 (March 29)	4.87 (4.25–5.58)	
	14 (April 5)	4.69 (4.10–5.37)	
	15 (April 12)	2.99 (2.62–3.42)	
Age group, years	18–45	Referent	< 0.0001
	45–65	1.30 (1.24–1.37)	
	65–75	1.38 (1.26–1.51)	
	75–85	1.46 (1.30–1.63)	
	≥ 85	1.60 (1.41–1.81)	
Sex	Female	Referent	< 0.0001
	Male	1.45 (1.31–1.60)	
Rural dwelling		0.96 (0.86–1.09)	0.56
Income quintile	1 (lowest)	1.09 (1.01–1.17)	
	2	0.96 (0.89–1.03)	
	3	1.05 (0.98–1.13)	
	4	1.01 (0.94–1.08)	
	5 (highest)	Referent	0.005
Canadian immigrant		1.53 (1.45–1.61)	< 0.0001
Frailty		1.13 (1.01–1.25)	0.03
Hospitalization or ED visits in 2019	0	Referent	< 0.0001
	1–2	0.77 (0.73–0.81)	
	3 +	0.54 (0.51–0.58)	
Hypertension		1.10 (1.04–1.17)	0.001
Diabetes		1.12 (1.05–1.20)	0.001
Coronary artery disease		0.78 (0.69–0.88)	< 0.0001
Heart failure		0.96 (0.81–1.14)	0.64
Stroke		1.13 (0.89–1.42)	0.31
Atrial fibrillation		0.87 (0.77–0.99)	0.03
Chronic kidney disease		0.83 (0.74–0.93)	0.001
HIV		0.64 (0.41–0.98)	0.04
Organ transplantation		0.61 (0.36–1.01)	0.05
Cancer		0.70 (0.61–0.81)	< 0.0001
Liver disease		0.66 (0.47–0.91)	0.01
Lung disease		0.83 (0.78–0.88)	< 0.0001
Influenza vaccination in 2019		0.98 (0.93–1.03)	0.41
Regional smoking rate, per 1%		1.00 (0.98–1.02)	0.65
Regional obesity rate, per 1%		0.98 (0.97–0.995)	0.003
Regional racial/ethnic diversity rate, per 1%		1.02 (1.01–1.03)	0.002
**Weeks of April 19–June 7, 2020**			
Week of testing (start date)	16 (April 19)	Referent	< 0.0001
	17 (April 26)	0.65 (0.62–0.70)	
	18 (May 3)	0.49 (0.46–0.52)	
	19 (May 10)	0.49 (0.46–0.53)	
	20 (May 17)	0.68 (0.64–0.73)	
	21 (May 24)	0.34 (0.32–0.36)	
	22 (May 31)	0.22 (0.20–0.24)	
	23 (June 7)	0.15 (0.14–0.17)	
Age group (years)	18–45	1.58 (1.40–1.80)	
	45–65	1.43 (1.27–1.62)	
	65–75	1.23 (1.08–1.40)	
	75–85	1.11 (0.97–1.28)	
	≥ 85	Referent	< 0.0001
Sex	Female	Referent	< 0.0001
	Male	1.67 (1.52–1.83)	
Rural dwelling		0.90 (0.79–1.04)	0.14
Income quintile	1 (lowest)	2.01 (1.87–2.16)	
	2	1.53 (1.43–1.65)	
	3	1.46 (1.36–1.57)	
	4	1.27 (1.17–1.37)	
	5 (highest)	Referent	< 0.0001
Canadian immigrant		1.82 (1.75–1.90)	< 0.0001
Frailty		0.94 (0.84–1.06)	0.32
Hospitalization or ED visits in 2019	0	Referent	< 0.0001
	1–2	0.97 (0.93–1.01)	
	3 +	0.82 (0.77–0.87)	
Hypertension		1.07 (1.01–1.12)	0.02
Diabetes		1.32 (1.24–1.40)	< 0.0001
Coronary artery disease		0.82 (0.73–0.93)	0.002
Heart failure		0.86 (0.71–1.05)	0.14
Stroke		1.05 (0.82–1.34)	0.71
Atrial fibrillation		0.92 (0.80–1.04)	0.17
Chronic kidney disease		0.70 (0.62–0.78)	< 0.0001
HIV		0.67 (0.48–0.95)	0.03
Organ transplantation		0.71 (0.42–1.20)	0.20
Cancer		0.53 (0.45–0.62)	< 0.0001
Liver disease		0.65 (0.47–0.90)	0.009
Lung disease		0.90 (0.85–0.95)	< 0.0001
Influenza vaccination in 2019		0.78 (0.74–0.82)	< 0.0001
Regional smoking rate, per 1%		0.99 (0.97–1.01)	0.25
Regional obesity rate, per 1%		1.01 (1.00–1.02)	0.30
Regional racial/ethnic diversity rate, per 1%		1.05 (1.03–1.06)	< 0.0001
**Weeks of June 14–December 27, 2020**			
Week of testing (start date)	24 (June 14)	Referent	< 0.0001
	25 (June 21)	1.18 (1.12–1.23)	
	26 (June 28)	0.88 (0.83–0.93)	
	27 (July 5)	0.78 (0.73–0.82)	
	28 (July 12)	0.81 (0.77–0.86)	
	29 (July 19)	0.77 (0.73–0.82)	
	30 (July 26)	0.71 (0.67–0.75)	
	31 (August 2)	0.65 (0.61–0.70)	
	32 (August 9)	0.65 (0.61–0.70)	
	33 (August 16)	0.66 (0.62–0.71)	
	34 (August 23)	0.74 (0.70–0.79)	
	35 (August 30)	0.76 (0.71–0.80)	
	36 (September 6)	0.96 (0.91–1.01)	
	37 (September 13)	1.08 (1.02–1.13)	
	38 (September 20)	1.20 (1.14–1.26)	
	39 (September 27)	1.35 (1.29–1.42)	
	40 (October 4)	1.42 (1.36–1.49)	
	41 (October 11)	1.75 (1.67–1.83)	
	42 (October 18)	1.97 (1.88–2.06)	
	43 (October 25)	2.07 (1.98–2.17)	
	44 (November 1)	2.43 (2.33–2.54)	
	45 (November 8)	2.74 (2.62–2.86)	
	46 (November 15)	2.61 (2.50–2.72)	
	47 (November 22)	2.81 (2.70–2.94)	
	48 (November 29)	2.96 (2.84–3.09)	
	49 (December 6)	2.96 (2.84–3.08)	
	50 (December 13)	3.28 (3.15–3.42)	
	51 (December 20)	4.01 (3.84–4.18)	
	52 (December 27)	5.62 (5.39–5.87)	
Age group (years)	18–45	1.09 (1.03–1.14)	
	45–65	1.04 (0.99–1.09)	
	66–75	0.87 (0.83–0.92)	
	76–85	0.85 (0.81–0.90)	
	≥ 85	Referent	< 0.0001
Sex	Female	Referent	< 0.0001
	Male	1.24 (1.20–1.27)	
Rural dwelling		1.03 (0.99–1.07)	0.12
Income quintile	1 (lowest)	1.64 (1.60–1.67)	
	2	1.45 (1.42–1.48)	
	3	1.39 (1.36–1.42)	
	4	1.17 (1.15–1.19)	
	5 (highest)	Referent	<0.0001
Canadian immigrant		1.99 (1.96–2.01)	< 0.0001
Frailty		0.97 (0.93–1.02)	0.25
Hospitalization or ED visits in 2019	0	Referent	< 0.0001
	1–2	1.06 (1.04–1.07)	
	3 +	0.86 (0.83–0.89)	
Hypertension		1.08 (1.07–1.10)	< 0.0001
Diabetes		1.33 (1.30–1.36)	< 0.0001
Coronary artery disease		0.92 (0.88–0.96)	< 0.0001
Heart failure		0.95 (0.88–1.03)	0.20
Stroke		0.90 (0.81–0.99)	0.04
Atrial fibrillation		0.86 (0.82–0.90)	< 0.0001
Chronic kidney disease		0.95 (0.91–0.98)	0.006
HIV		0.86 (0.75–0.98)	0.02
Organ transplantation		1.01 (0.85–1.20)	0.92
Cancer		0.66 (0.63–0.69)	< 0.0001
Liver disease		0.66 (0.58–0.75)	< 0.0001
Lung disease		1.00 (0.98–1.02)	0.95
Influenza vaccination in 2019		0.75 (0.74–0.77)	< 0.0001
Regional smoking rate, per 1%		0.99 (0.98–0.99)	< 0.0001
Regional obesity rate, per 1%		1.00 (1.00–1.01)	0.03
Regional racial/ethnic diversity rate, per 1%		1.04 (1.03–1.05)	< 0.0001

Odds ratios were determined from a multivariable logistic regression model that incorporated PHU-specific random effects.

For continuous variables, results are per 1-unit increase in rate.

In the early stages of modelling, there was significant heterogeneity in the overall model between age and week of testing (*P*-interaction < 0.0001), hence results are presented stratified prior to and following the peak of the first wave of the pandemic until the end of 2020.

*CI* confidence intervals, *ED* emergency department, *HIV* human immunodeficiency virus, *OR* odds ratio.

Immediately following the peak of the first wave of the pandemic, the likelihood of laboratory-confirmed SARS-CoV-2 infection across age groups in community-dwelling individuals reversed (Supplemental Fig. 4). Thereafter, the oldest individuals aged ≥ 85 years had the lowest likelihood of SARS-CoV-2 infection (Table 2), with a progressive increase in infection risk as age declined compared with individuals aged ≥ 85 years (age 75–85, OR 1.11, 95% CI 0.97–1.28; age 65–75, OR 1.23, 95% CI 1.08–1.40; age 45–65, OR 1.43, 95% CI 1.27–1.62; and age 18–45, OR 1.58, 95% CI 1.40–1.80). Additionally, there was a progressive increased risk of SARS-CoV-2 infection across all lower quintiles of neighborhood income compared with the highest quintile (quintile 1, OR 2.01, 95% CI 1.87–2.16; quintile 2, OR 1.53, 95% CI 1.43–1.65; quintile 3, OR 1.46, 95% CI 1.36–1.57; quintile 4, OR 1.27, 95% CI 1.17–1.37). Other independent risk factors included male sex (OR 1.67, 95% CI 1.52–1.83), residing in more racially/ethnically diverse communities (OR per 1% increase 1.05, 95% CI 1.03–1.06), immigration to Canada (OR 1.82, 95% CI 1.75–1.90), history of hypertension (OR 1.07, 95% CI 1.01–1.12), and history of diabetes (OR 1.32, 95% CI 1.24–1.40).

Results through the remainder of 2020 are reported in Table 2. Independent risk factors continued to be younger individuals (age 18–45, OR 1.09, 95% CI 1.03–1.14), who had the highest likelihoods of SARS-CoV-2 infection compared with older age groups (age 66–75, OR 0.87, 95% CI 0.83–0.92; and age 76–85, OR 0.85, 95% CI 0.81–0.90). There continued to be a progressive increase in the risk of infection in the lower quintiles of neighborhood income (quintile 1, OR 1.64, 95% CI 1.60–1.67; quintile 2, OR 1.45, 95% CI 1.42–1.48; quintile 3, OR 1.39, 95% CI 1.36–1.42; quintile 4, OR 1.17, 95% CI 1.15–1.19). Other independent risk factors included male sex (OR 1.24, 95% CI 1.20–1.27), residing in more racially/ethnically diverse communities (OR per 1% increase 1.04, 95% CI 1.03–1.05), immigration to Canada (OR 1.99, 95% CI 1.96–2.01), history of hypertension (OR 1.08, 95% CI 1.07–1.10), and history of diabetes (OR 1.33, 95% CI 1.30–1.36).

**Figure 2:**
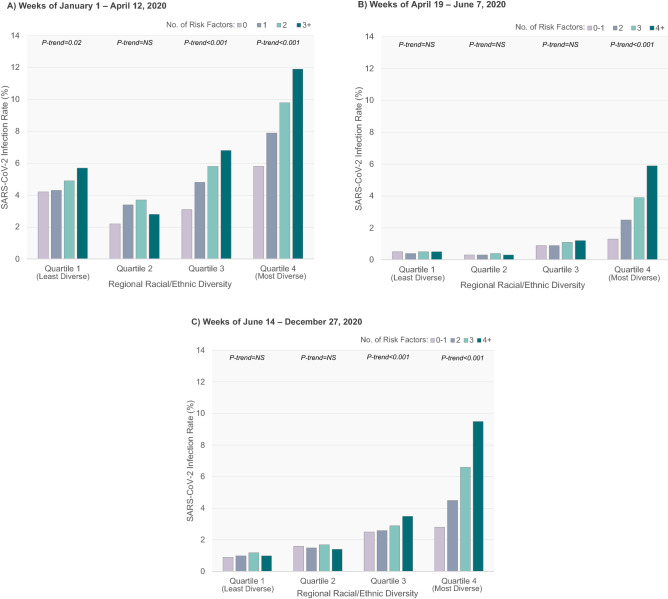
SARS-CoV-2 infection rates by number of risk factors and degree of regional racial/ethnic diversity, prior to (**A** Weeks of January 1–April 12, 2020) and following (**B** Weeks of April 19–June 7, 2020 and **C** Weeks of June 14–December 27, 2020) the peak of the first wave of the pandemic in Ontario, Canada. Individuals were classified according to the number of potential risk factors present. Risk factors prior to the first wave were comprised of: male sex, age > 45 years, lowest quintile of neighborhood income, Canadian immigrant, history of frailty, hypertension, and diabetes; following the first wave: male sex, age < 85 years, neighborhood income quintiles 1–4, Canadian immigrant, hypertension, and diabetes. Region was categorized by quartiles of community racial/ethnic diversity rate.

The absolute and relative risk of laboratory-confirmed SARS-CoV-2 infection among community-dwelling individuals according to the number of independent risk factors identified above (e.g., age category, male sex, residing in a lower income neighborhood, Canadian immigrant status, hypertension, diabetes, and, prior to the peak of the pandemic, a history of frailty), degree of regional racial/ethnic diversity, and time period are shown in Fig. 2 and Table 3. Prior to the peak of the pandemic, SARS-CoV-2 infection rates were generally greater across communities with more racial/ethnic diversity and among individuals with a higher number of risk factors such that individuals living in the most racially/ethnically diverse communities without any other risk factors had a similar rate of infection as individuals living in the least racially/ethnically diverse communities with 3 or more risk factors (Fig. 2a). Individuals with 1, 2, or ≥ 3 risk factors had progressively higher odds of SARS-CoV-2 infection compared with individuals without risk factors across regions of racial/ethnic diversity.

**Table 3:** Rates and odds ratios of SARS-CoV-2 infection by number of risk factors and degree of regional racial/ethnic diversity, prior to (weeks of January 1 to April 12, 2020) and following (weeks of April 19 to June 7, 2020; weeks of June 14 to December 27, 2020) the peak of the first wave of the pandemic among community-dwelling individuals in Ontario, Canada.

**Weeks of January 1–April 12, 2020**				
Racial/ethnic diversity quartile 1 (≤ 2.5%; least diverse)	COVID-19 positive (n = 390) (4.8%)	Total (n = 8123)	OR (95% CI)	*P*-trend
**Number of risk factors**				
0	64 (4.2%)	1518	Referent	0.017
1	105 (4.3%)	2451	1.02 (0.74–1.40)	
2	98 (4.9%)	1989	1.18 (0.85–1.63)	
3 +	123 (5.7%)	2165	1.37 (1.004–1.87)	

Racial/ethnic diversity quartile 2 (2.6–4.0%)	COVID-19 positive (n = 348) (3.1%)	Total (n = 11,333)	OR (95% CI)	*P*-trend
**Number of risk factors**				
0	49 (2.2%)	2263	Referent	0.308
1	118 (3.4%)	3445	1.60 (1.14–2.25)	
2	99 (3.7%)	2676	1.74 (1.23–2.46)	
3 +	82 (2.8%)	2949	1.29 (0.90–1.85)	

Racial/ethnic diversity quartile 3 (4.1–17.6%)	COVID-19 positive (n = 1184) (5.5%)	Total (n = 21,560)	OR (95% CI)	*P*-trend
**Number of risk factors**				
0	142 (3.1%)	4556	Referent	< 0.001
1	336 (4.8%)	6960	1.58 (1.29–1.93)	
2	307 (5.8%)	5337	1.90 (1.55–2.32)	
3 +	399 (6.8%)	5891	2.26 (1.86–2.75)	

Racial/ethnic diversity quartile 4 (≥ 17.7%; most diverse)	COVID-19 positive (n = 7071) (9.3%)	Total (n = 76,270)	OR (95% CI)	*P*-trend
**Number of risk factors**				
0	704 (5.8%)	12,052	Referent	< 0.001
1	1684 (7.9%)	21,390	1.38 (1.26–1.51)	
2	1897 (9.8%)	19,320	1.76 (1.61–1.92)	
3 +	2786 (11.9%)	23,508	2.17 (1.99–2.36)	

**Weeks of April 19–June 7, 2020**				
Racial/ethnic diversity quartile 1 (≤ 2.5%; least diverse)	COVID-19 positive (n = 165) (0.5%)	Total (n = 36,173)	OR (95% CI)	*P*-trend
**Number of risk factors**				
0–1	15 (0.5%)	2785	Referent	0.32
2	61 (0.4%)	15,899	0.71 (0.40–1.25)	
3	59 (0.5%)	11,772	0.93 (0.53–1.64)	
4 +	30 (0.5%)	5717	0.97 (0.52–1.81)	

Racial/ethnic diversity quartile 2 (2.6–4.0%)	COVID-19 positive (n = 204) (0.3%)	Total (n = 59,811)	OR (95% CI)	*P*-trend
**Number of risk factors**				
0–1	15 (0.3%)	4509	Referent	0.63
2	79 (0.3%)	25,811	0.92 (0.53–1.60)	
3	80 (0.4%)	19,524	1.23 (0.71–2.14)	
4 +	30 (0.3%)	9967	0.91 (0.49–1.68)	

Racial/ethnic diversity quartile 3 (4.1–17.6%)	COVID-19 positive (n = 924) (1.0%)	Total (n = 91,689)	OR (95% CI)	*P*-trend
**Number of risk factors**				
0–1	73 (0.9%)	7813	Referent	< 0.001
2	330 (0.9%)	37,713	0.94 (0.73–1.21)	
3	334 (1.1%)	30,886	1.16 (0.90–1.50)	
4 +	187 (1.2%)	15,277	1.31 (1.001–1.73)	

Racial/ethnic diversity quartile 4 (≥ 17.7%; most diverse)	COVID-19 positive (n = 10,238) (3.8%)	Total (n = 272,120)	OR (95% CI)	*P*-trend
**Number of risk factors**				
0–1	258 (1.3%)	19,583	Referent	< 0.001
2	2128 (2.5%)	86,572	1.89 (1.66–2.15)	
3	3928 (3.9%)	99,931	3.07 (2.70–3.48)	
4 +	3924 (5.9%)	66,034	4.73 (4.17–5.37)	

**Weeks of June 14–December 27, 2020**				
Racial/ethnic diversity quartile 1 (≤ 2.5%; least diverse)	COVID-19 positive (n = 1757) (1.1%)	Total (n = 165,328)	OR (95% CI)	*P*-trend
**Number of risk factors**				
0–1	119 (0.9%)	13,182	Referent	0.202
2	706 (1.0%)	69,152	1.13 (0.93–1.38)	
3	696 (1.2%)	58,292	1.33 (1.09–1.61)	
4 +	236 (1.0%)	24,702	1.06 (0.85–1.32)	

Racial/ethnic diversity quartile 2 (2.6–4.0%)	COVID-19 positive (n = 4106) (1.6%)	Total (n = 263,851)	OR (95% CI)	*P*-trend
**Number of risk factors**				
0–1	326 (1.6%)	20,488	Referent	0.847
2	1677 (1.5%)	111,082	0.95 (0.84–1.07)	
3	1514 (1.7%)	91,370	1.04 (0.92–1.18)	
4 +	589 (1.4%)	40,911	0.90 (0.79–1.04)	

Racial/ethnic diversity quartile 3 (4.1–17.6%)	COVID-19 positive (n = 14,271) (2.8%)	Total (n = 503,567)	OR (95% CI)	*P*-trend
**Number of risk factors**				
0–1	1217 (2.5%)	48,229	Referent	< 0.001
2	5374 (2.6%)	207,764	1.03 (0.96–1.09)	
3	5112 (2.9%)	173,600	1.17 (1.10–1.25)	
4 +	2568 (3.5%)	73,974	1.39 (1.30–1.49)	

Racial/ethnic diversity quartile 4 (≥ 17.7%; most diverse)	COVID-19 positive (n = 102,156) (6.2%)	Total (n = 1,656,744)	OR (95% CI)	*P*-trend
**Number of risk factors**				
0–1	3869 (2.8%)	140,562	Referent	< 0.001
2	25,068 (4.5%)	558,357	1.66 (1.60–1.72)	
3	39,653 (6.6%)	603,726	2.48 (2.40–2.57)	
4 +	33,566 (9.5%)	354,099	3.70 (3.58–3.83)	

Number of potential risk factors prior to the first wave: male sex, age > 45 years, lowest quintile of neighborhood income, Canadian immigrant, history of frailty, hypertension, and diabetes; following the first wave: male sex, age < 85 years, neighborhood income quintiles 1-4, Canadian immigrant, hypertension, and diabetes.

Region was categorized by quartiles of community racial/ethnic diversity rate.

Following the peak of the first wave of the pandemic in mid-April and continuing for the remainder of 2020, infection rates declined across Ontario, however there was less impact in regions with higher degrees of racial/ethnic diversity. Additionally, while accumulation of more risk factors remained associated with a higher risk of SARS-CoV-2 infection in the most racially/ethnically diverse communities, the risk factors now included all age groups < 85 years (Fig. 2b and c; Table 3). Immediately following the first-wave peak, individuals living in the most racially/ethnically diverse communities with 2, 3, or ≥ 4 risk factors had ORs of 1.89, 3.07, and 4.73 for SARS-CoV-2 infection compared to lower risk individuals in their community with 0–1 risk factors. In contrast, in the least racially/ethnically diverse communities, there was little to no gradient in infection rates across risk strata. For the remainder of 2020, although the absolute infection rates increased across all four quartiles in comparison to immediately following the first-wave peak, the ORs for SARS-CoV-2 infection among individuals living in the most racially/ethnically diverse communities with 2, 3, or ≥ 4 risk factors remained significantly elevated at 1.66, 2.48, and 3.70, with little gradient in infection rates across risk strata among the least racially/ethnically diverse communities.

## Discussion

We observed three dynamic factors during the first wave and first year of the pandemic associated with laboratory-confirmed SARS-CoV-2 infection among community-dwelling individuals in Ontario that merit consideration for risk determination, response planning, and health policy. First, we detected significant time-varying heterogeneity in the association between age and the odds of SARS-CoV-2 infection. Initially, an incremental increase in age was independently associated with a higher odds of SARS-CoV-2 infection, a biologic risk factor representing a more vulnerable population susceptible to viral infection. After the implementation of public health measures in late March 2020, the association reversed with the odds of SARS-CoV-2 infection increasing across progressively younger age groups compared with community-dwelling individuals 85 years and older. Second, the number of independent risk factors was associated with a stepwise increase in the odds of SARS-CoV-2 infection. Across Ontario, there was an initial increased odds of infection associated with the presence of more clinical risk factors (such as diabetes and hypertension), which was accentuated in regions with higher racial/ethnic diversity. After public health measures were implemented, the absolute and relative risk associated with the accumulation of risk factors diminished overall. However, among the regions of Ontario with the highest rates of racial/ethnic diversity, the relative odds of infection associated with a higher number of risk factors remained present. Third, we observed that the risk of SARS-CoV-2 infection was associated with higher regional racial/ethnic diversity. Following public health measures, regional racial/ethnic diversity remained independently associated with higher odds of SARS-CoV-2 infection, though absolute rates were reduced.

We analyzed cumulative risk stratified across quartiles of community racial/ethnic diversity to ascertain to what degree the presence of an increasing burden of sociodemographic and clinical risk factors was predictive of laboratory-confirmed SARS-CoV-2 infection independent of residential factors. Regions of Ontario with the highest racial/ethnic diversity correlate with the largest sized communities and the highest neighborhood density, household crowding, and deprivation^37^. Lower infection rates in the period following the pandemic’s initial peak correlated with implementation of national and provincial restrictions, including restrictions in international travel and closure of schools and non-essential businesses. These data suggest that these broad public health interventions are associated with changes in the likelihood of infection by age, but had little impact on the other risk factors driving virus transmission. These risk factors may represent characteristics of individuals at higher risk of exposure, those working in essential services or living in densely populated housing, or both, and not represent a higher biologic susceptibility to infection. Systemic and structural inequities in these determinants of health are likely associated with higher residential and occupational risk of viral transmission and reduced ability to comply with isolation orders. These data may help inform policy to protect more vulnerable populations including essential workers, such as the implementation of paid sick leave, targeted screening in heavily impacted neighborhoods, and “wrap-around” services, to ensure those infected have a safe place to isolate and not infect others in the home.

There are several strengths of our study. This is the first North American population-based analysis of the cumulative effect of clinical and sociodemographic risk factors stratified by community racial/ethnic diversity and time. This approach unmasked considerable differences in the age-related susceptibility to SARS-CoV-2 infection before and after the peak of the first wave of the pandemic that continued until the end of 2020, and the residual risk that remained despite broad public health measures in large, urban, racially/ethnically diverse regions of the province most impacted by COVID-19. These findings have implications for the effectiveness of current public health measures to restrict SARS-CoV-2 infection, particularly among young, male, immigrants living in lower socioeconomic neighborhoods who are likely working or living in conditions not conducive to adherence to these measures. There are important limitations to acknowledge as well. Access to testing for SARS-CoV-2 infection varied over time, including restriction of testing during the earlier periods to the highest risk patients; while some patients with severe COVID-19 illness may also have died before testing. Over time, testing capacity increased and broader testing occurred. As a result, our analysis was focused on individuals that underwent SARS-CoV-2 testing to reduce ascertainment bias and among whom our results apply. In addition, a small number of hospital-based SARS-CoV-2 test results were not available for this analysis, but this low percentage of missingness would not be expected to materially impact the results of available data. Since we did not have individual-level data on weight, smoking status, income, and race/ethnicity, we relied on community-level variables as proxies, which underestimates the extent of socioeconomic and racial/ethnic inequities in SARS-CoV-2 infection. These analyses would have also benefited from incorporating other individual-level socioeconomic data that are currently not routinely captured by the province, such as data on missed workdays and contributing reasons (e.g., quarantine, isolation, etc.), which may relate to infection. During both time periods, a number of chronic conditions were significantly associated with a lower risk of laboratory-confirmed SARS-CoV-2 infection, likely reflecting collider/screening bias among asymptomatic patients undergoing regular care^38^. Finally, the analyses presented here do not discuss the downstream clinical impact of SARS-CoV-2 infection as these will be reported in a subsequent paper.

Despite the dramatic impact of a provincial lockdown, following the peak of the initial wave of the pandemic in early April, the highest likelihood of SARS-CoV-2 infection emerged among clusters of people represented by younger age, male sex, individuals that immigrated to Canada, with hypertension or diabetes, and residing in the most racially/ethnically diverse, urban, most socioeconomically disadvantaged communities of Ontario. Further efforts appear necessary to reduce the risk of SARS-CoV-2 infection among the highest risk individuals residing in these communities.

## Supplementary Information

Supplementary Information.

## Data Availability

The dataset from this study is held securely in coded form at ICES. While legal data sharing agreements between ICES and data providers (e.g., healthcare organizations and government) prohibit ICES from making the dataset publicly available, access may be granted to those who meet pre-specified criteria for confidential access, available at www.ices.on.ca/DAS (email: das@ices.on.ca).
