# Supplementary material for: Clinical risk, sociodemographic factors, and SARS-CoV-2 infection over time in Ontario, Canada
Source: Sci Rep. 2022 Jun 24;12:10534. doi: 10.1038/s41598-022-13598-z (PMC9232511; doi:10.1038/s41598-022-13598-z)
Supplement: Supplementary file 1 — Supplementary Information. [file 41598_2022_13598_MOESM1_ESM.pdf]

Clinical Risk, Sociodemographic Factors, and SARS-CoV-2 Infection Over Time in Ontario, Canada

Short Title: Sociodemographic and Clinical Risk of COVID-19 Over Time in Ontario

Authors:
Jacob A. Udell, MD, MPH [1,2,3,4,5*]
Bahar Behrouzi, MSc [1,2,4,5]
Atul Sivaswamy, MSc [1]
Anna Chu, MHSc [1]
Laura E. Ferreira-Legere, RN, MScN [1]
Jiming Fang, PhD [1]
Shaun G. Goodman, MD, MSc [5,6,7]
Justin A. Ezekowitz, MBBCh, MSc[7,8,9]
Kevin R. Bainey, MD, MSc [7,8,9]
Sean van Diepen, MD, MSc [7,8,9]
Padma Kaul, PhD [7,8,9]
Finlay A. McAlister, MD, MSc [7,8,9]
Isaac I. Bogoch, MD, MPH [5, 10]
Cynthia A. Jackevicius, BSCPhm, PharmD, MSc [1,4,11]
Husam Abdel-Qadir, MD, PhD [1,2,3,4,5]
Harindra C. Wijeysundera, MD, PhD [1,4,5,12]
Dennis T. Ko, MD, MSc [1,4,5,12]
Peter C. Austin, PhD [1,4]
Douglas S. Lee, MD, PhD, FRCPC [1,3,4,5]

Author Affiliations:
(1) ICES, Toronto, Canada
(2) Cardiovascular Division, Department of Medicine, Women's College Hospital, Toronto, Canada
(3) Peter Munk Cardiac Centre, University Health Network, Toronto, Canada
(4) Institute of Health Policy, Management, and Evaluation, University of Toronto, Toronto, Canada
(5) Department of Medicine, Temerty Faculty of Medicine, University of Toronto, Toronto, Canada
(6) Division of Cardiology, St. Michael's Hospital, Toronto, Canada
(7) Canadian VIGOUR Centre, University of Alberta, Edmonton, Canada
(8) Department of Medicine, Faculty of Medicine & Dentistry, University of Alberta, Edmonton, Canada
(9) Department of Critical Care Medicine and Division of Cardiology, Department of Medicine, University of Alberta, Edmonton, Canada
(10) Divisions of General Internal Medicine and Infectious Diseases, University Health Network, Toronto, Canada
(11) Western University of Health Sciences, Pomona, CA
(12) Schulich Heart Centre, Sunnybrook Health Sciences Centre, Toronto, Canada

Address for Correspondence: Jacob A. Udell, Peter Munk Cardiac Centre, Toronto General Hospital and Cardiovascular Division, Women's College Hospital, University of Toronto, 76 Grenville Street, Toronto, ON M5S 1B2, Canada.

Online-Only Supplement

Table of Contents

Supplemental Figure 1. Timeline of Ontario/Canada's Pandemic Response to COVID-19 Until December 31, 2020

Supplemental Figure 2. Study eligibility flow chart for the A) first and B) second halves of 2020 in Ontario, Canada

Supplemental Figure 3. Distribution of Regional Racial/Ethnic Diversity in Ontario as a Percentage of Total Population by Public Health Unit

Supplemental Figure 4. Weekly number of community-dwelling individuals tested for SARS-CoV-2, and share of tests that were positive, stratified by age group during 2020, prior to (A. Weeks of January 1 – April 12) and following (B. Weeks of April 19 – June 7 and C. Weeks of June 14 – December 27) the peak of the first wave of the pandemic in Ontario, Canada/

Supplemental Table 1. Administrative Health Data Codes Defining Baseline Clinical Diagnoses

Supplemental Table 2. Baseline Characteristics of Community-Dwelling Individuals with and without SARS-CoV-2 Infection in Ontario, Canada between the weeks of June 14 to December 27, 2020

Supplemental References

Supplemental Figure 1. Timeline of Ontario/Canada's Pandemic Response to COVID-19 Until December 31, 2020[1,2]

	January
1/30	Canada issues a travel advisory to avoid all non-essential travel to China, avoid all travel to Hubei province
	February
	March
3/3	Canada issues a travel advisory to avoid all non-essential travel to Iran
3/5	Canada issues a travel advisory to avoid all travel to Iran<br>Ontario expands SARS-CoV-2 testing
3/9	Public Health Agency of Canada issues a travel advisory to avoid all travel on cruise ships
3/11	Public Health Agency of Canada issues guidelines on when to cancel mass gatherings<br>WHO declares a global pandemic
3/12	Ontario ordered all public schools to close, remains closed for duration of the school year
3/13	Ontario recommends immediate cancellation of all events >250 people and advises against travel outside Canada<br>Ontario universities begin moving to online education
3/14	Ontario urged long-term care residences to bar all but "essential visitors"
3/16	Canada closes its border to non-citizens/residents
3/17	Ontario declares a state of emergency and bans public events of >50 people, closure of all indoor facilities, bars, and restaurants
3/18	Canada/US closes its border to non-essential travel
3/24	Ontario orders all non-essential workplaces/businesses to close
3/30	Ontario declares closure of all outdoor recreation amenities, advises all people over 70 or with compromised immune systems to self-isolate
	April
4/6	Canada advises people to wear non-medical face masks to reduce transmission
4/9	Ontario broadened its testing criteria to include all symptomatic people who are hospital inpatients, long-term care residents, health care workers and caregivers, first responders, and residents of remote or Indigenous communities
4/27	Ontario releases plan for phased reopening
	May
5/9	Ontario allows some seasonal businesses and construction projects to reopen
5/16	Ontario begins phased reopening
5/20	Canada advises wearing masks when unable to distance
5/27	Ontario extends pandemic restrictions; keeps playgrounds, pools, restaurants closed; outdoor gatherings limited to 5 persons
5/29	Ontario expands testing strategy; targets campaigns to workers in key sectors, communities with outbreaks
	June
6/1	Ontario reopens provincial parks, drive-in movie theatres, batting cages
6/7	24 of Ontario's 34 public health units start second phase of easing pandemic restrictions (i.e., outdoor gatherings limited to 10, opening of outdoor dining and recreational facilities
6/17	Ontario extends emergency orders until June 30
6/22	Toronto and Peel region start second phase of easing pandemic restrictions
6/24	Ontario extends emergency orders until July 15
	July
7/6	All of Ontario is in second phase of easing pandemic restrictions
7/16	Ontario extends emergency orders until July 29
7/24	Hamilton, select large regions, select counties enter third phase of easing pandemic restrictions
7/31	Toronto and Peel region enter third phase of easing pandemic restrictions
	August
8/14	Windsor-Essex enters third phase of easing pandemic restrictions
8/20	Ontario extends emergency orders until September 22

September	
9/8	Health Minister announces pause in loosening restrictions
9/17	Limits on private outdoor (25 persons) and indoor (10 persons) gatherings in Toronto, Peel region, Ottawa; while limits eased in the rest of Ontario
9/25	Ontario closes strip clubs; orders food/drink businesses to close between 12AM-5AM; Pharmacies in greater Toronto area, Huntsville, and Ottawa offer testing to at-risk, asymptomatic persons
October	
10/1	Ontario announces new screening guidelines for daycares, schools
10/2	Ontario announces mask policy; restricts patrons in bars, restaurants, nightclubs, event venues, gyms
10/9	Closure of indoor dining, gyms, theatres, event venues in Toronto, Peel region, Ottawa
November	
11/3	Ontario introduces 5-tiered, colour-coded system for regulating regions based on pandemic impact (i.e., Green – prevention/minimum measures, Yellow – protection measures, Orange – restriction measures, Red – control measures, Grey – lockdown/maximum measures)
11/10	Toronto enters Red zone
11/20	Ontario extends emergency orders until December 21
11/23	Toronto and Peel region enter Grey zone (lockdown)
11/23	Canada extends ban on international travelers for non-essential purposes (except the US)
December	
12/7	Ontario announces 3-part vaccination rollout plan; prioritizes seniors, caregivers, healthcare workers
12/9	Canada approves use of Pfizer vaccine
12/11	York region and Windsor-Essex enter Grey zone (lockdown)
12/14	Ontario announces access to testing no longer free for international travel
12/21	Canada halts flights from the UK due to variant of concern
12/23	Canada approves use of Moderna vaccine
12/26	Ontario-wide lockdown begins (4 weeks in southern regions; 2 weeks in northern regions)
12/28	Ontario announces administration of 13,200 vaccines
12/30	Canada announces international air travelers must provide proof of negative PCR test on arrival, followed by mandatory 14-day quarantine

Supplemental Figure 2. Study eligibility flow chart for the A) first and B) second halves of 2020 in Ontario, Canada

A) First half of 2020

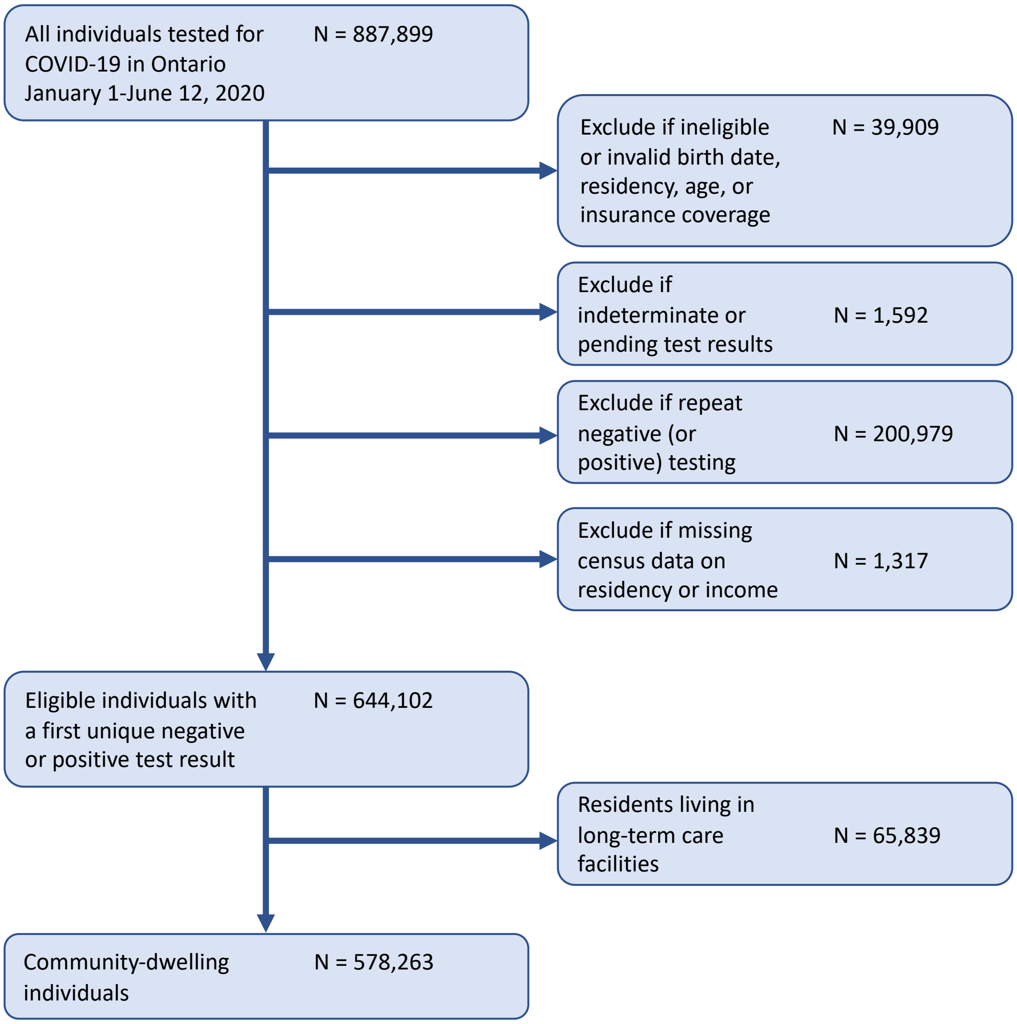

B) Second half of 2020

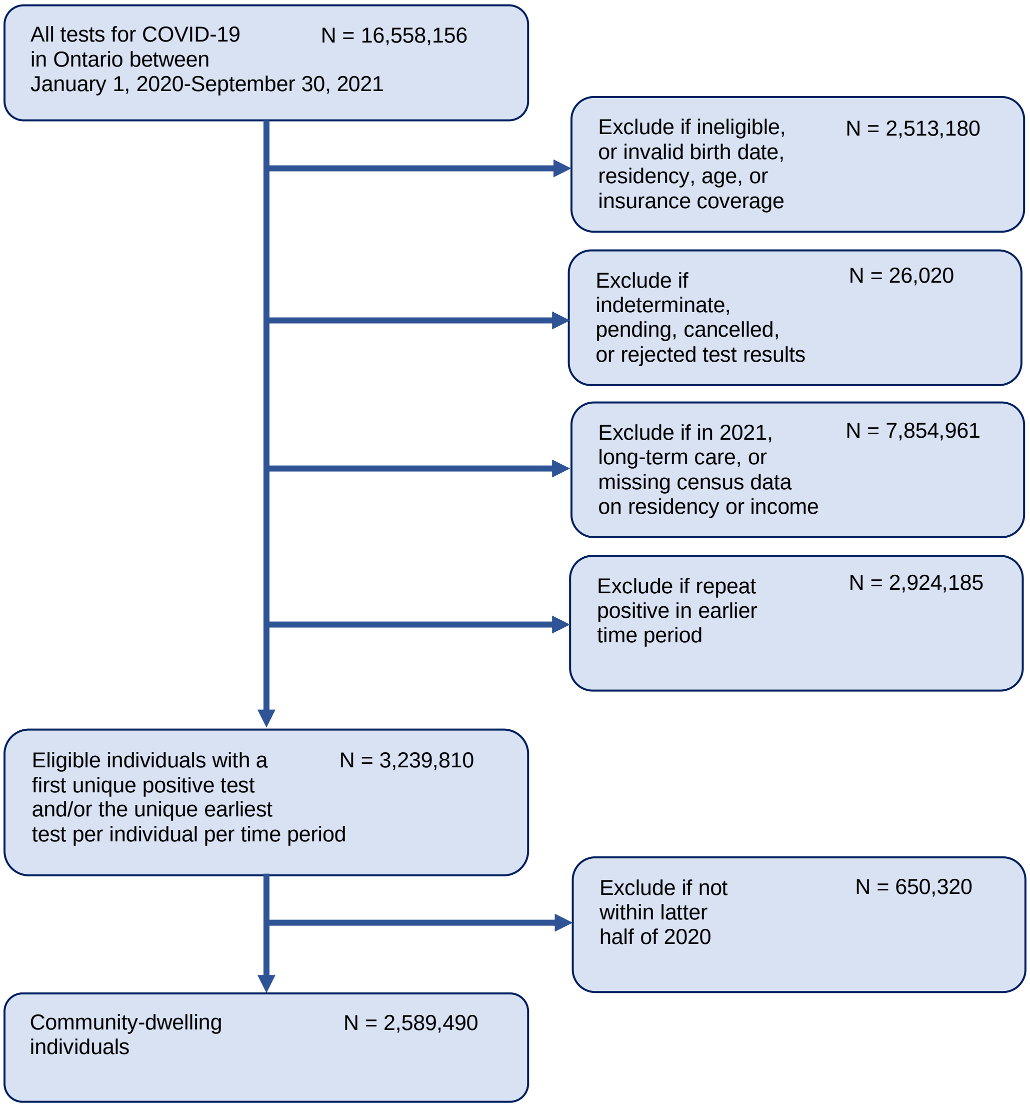

Supplemental Figure 3. Distribution of Regional Racial/Ethnic Diversity in Ontario as a Percentage of Total Population by Public Health Unit

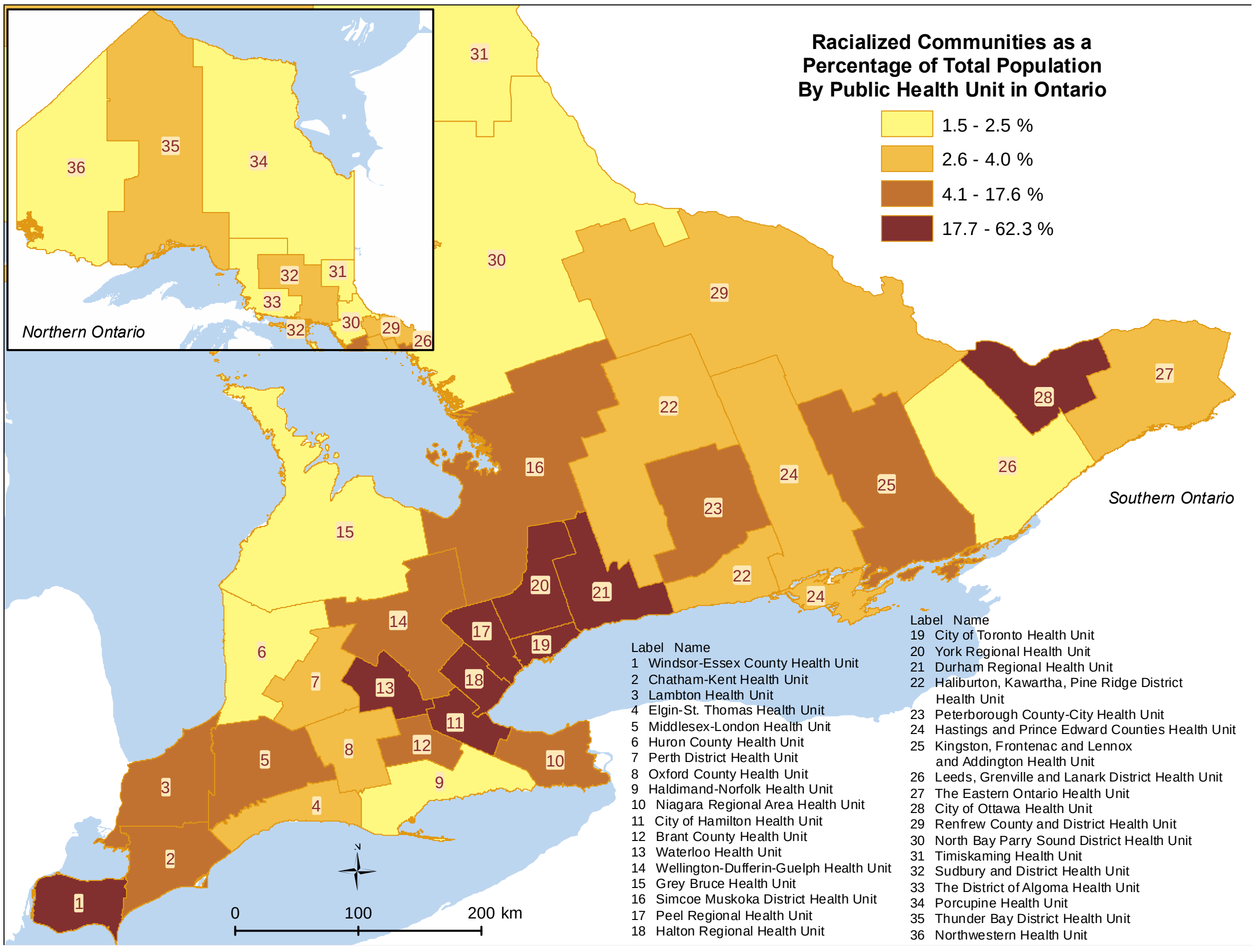

The percentage of racialized communities categorized by public health unit (PHU). PHUs were divided into quartiles; the median rate was 4.0% (interquartile range, 2.5-17.6%). Racial/ethnic diversity was defined as the regional visible minority proportion of individuals who self-identified as Black, South Asian, Chinese, Filipino, Latin American, Arab, Southeast Asian, West Asian, Korean and Japanese according to 2016 Census data. This map was constructed at ICES using ArcGIS Desktop version 10.7 by Esri (https://www.esri.com/en-us/arcgis/about-arcgis/overview).

Supplemental Figure 4. Weekly number of community-dwelling individuals tested for SARS-CoV-2, and share of tests that were positive, stratified by age group during 2020, prior to (A. Weeks of January 1 – April 12) and following (B. Weeks of April 19 – June 7 and C. Weeks of June 14 – December 27) the peak of the first wave of the pandemic in Ontario, Canada

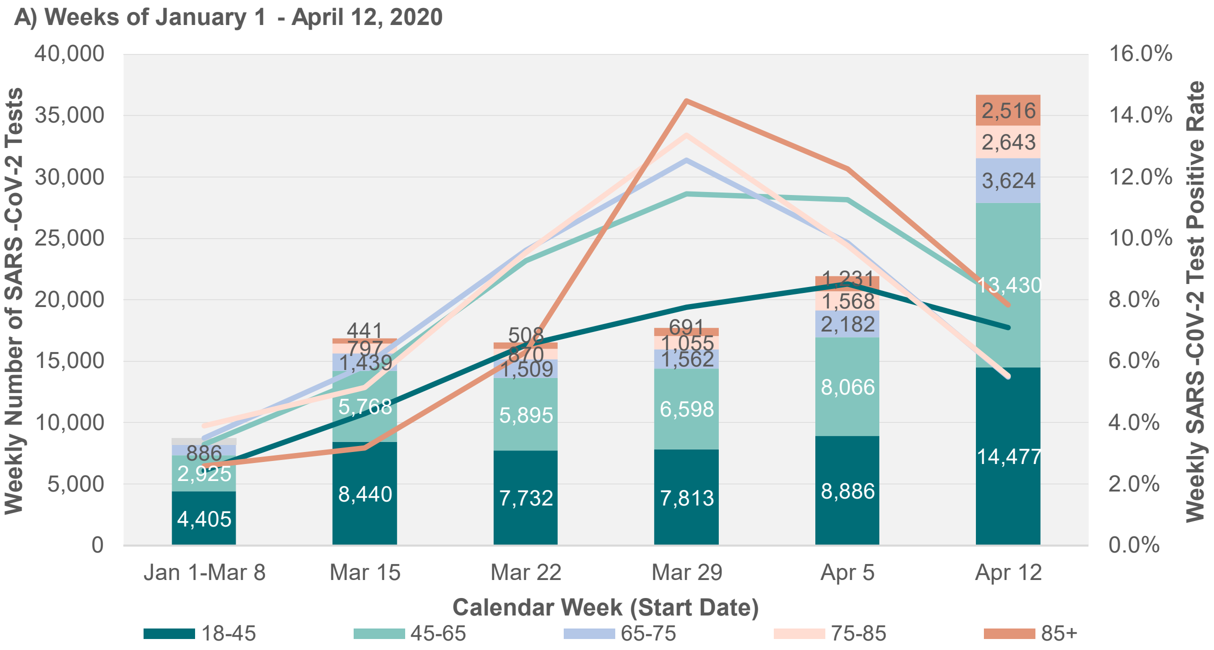

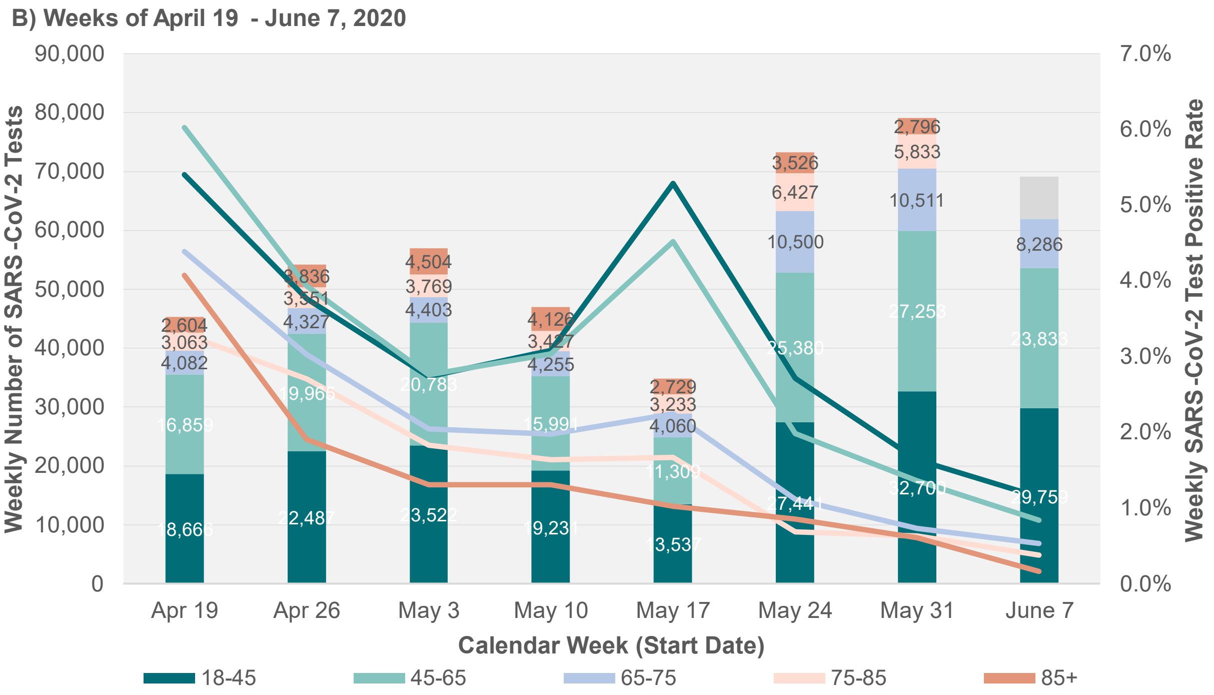

C) Weeks of June 14 - December 27, 2020

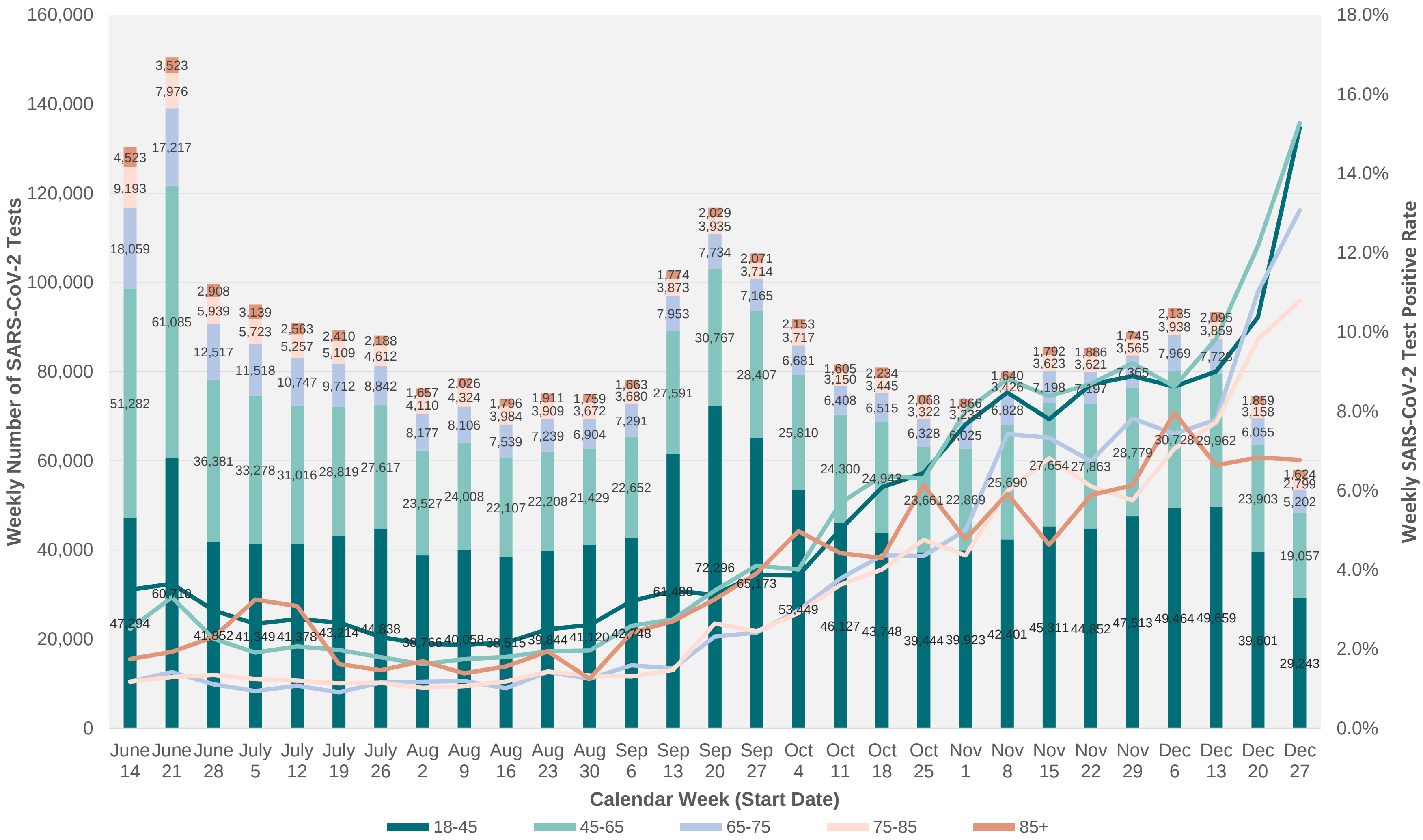

Time represented by the start of the calendar week with the weeks of January 1 through March 8 consolidated given low initial test counts. Age groups: 18-45 years (dark green); 45-65 years (light green); 65-75 years (light blue); 75-85 years (pink); 85 years and older (orange). The number of weekly counts attributable to an age group is represented in each stacked column (primary y-axis). The weekly positive test rate attributable to an age group is represented in each line graph (secondary y-axis). The share of positive test results among younger community-dwelling patients (18-65 years) overcoming those of older community-dwelling patients (65 years and older) during the first wave of the pandemic in Ontario occurred by the week of April 12, 2020. Ages 75 and up are combined to suppress small cells for weeks Jan 1 – March 8 and June 7, per ICES' reidentification risk assessment procedures.

Supplemental Table 1. Administrative Health Data Codes Defining Baseline Clinical Diagnoses

Variable	Database	Codes	Lookback Period
Prior number of hospitalizations or emergency department (ED) visits	CIHI-DAD<br>NACRS		From January 1st, 2019 to December 31st, 2019.
Coronary artery disease (CAD): defined as myocardial infarction, percutaneous or surgical coronary revascularization	CIHI-DAD, CIHI-SDS	ICD-10 diagnostic codes: I21, I22<br>CCP codes: 4802, 4803, or 481<br>CCI codes: 1IJ50, 1IJ57GQ, 1IJ54, 1IJ76	5 years prior
Heart failure (HF)	CIHI-DAD	ICD-10 diagnostic codes: I50	5 years prior
Stroke	CIHI-DAD	ICD-10 diagnostic codes: I60, I61, I63, I64, H341 (excluding I63.3)	5 years prior
Liver disease	CIHI-DAD	Mild: ICD-10 diagnostic codes: B18, K700-K703, K709, K713-K715, K717, K73, K74, K760, K762-K764, K768, K769, Z944<br><br>Moderate/severe: ICD-10 diagnostic codes: I850, I859, I864, I982, K704, K711, K721, K729, K765, K766, K767	5 years prior
Lung disease: defined as pneumonia, respiratory tuberculosis, asthma, or chronic obstructive pulmonary disease (COPD)	OHIP<br><br>NACRS, CIHI-DAD<br><br>NACRS, CIHI-DAD<br><br>OHIP<br><br>CIHI-DAD<br><br>An ICES-specific asthma database was used to identify patients ever diagnosed with asthma, based on ≥2 ambulatory	Pneumonia: OHIP diagnostic code: 486 (excluding claims associated with the fee codes G538, G539, G840-G848, G590, G591 or G700 [administration of vaccinations])<br>ICD-10 diagnostic codes: J10.0, J11.0 or J12-J18[5]<br><br>Respiratory tuberculosis<br>ICD-10 diagnostic codes: A15-A16<br><br>Asthma<br>OHIP diagnostic code: 493<br><br>ICD-10 diagnostic codes: J45, J46<br>ICD-9 diagnostic code: 493	5 years prior<br><br>5 years prior<br><br>Ever

	care visits and/or ≥ 1 hospitalizations[3]<br><br>OHIP<br><br>CIHI-DAD<br><br>An ICES-specific COPD database was used to identify patients ever diagnosed with COPD, based on ≥ 1 hospitalizations and/or ≥ 1 ambulatory care visits. The algorithm for identifying these patients has only been validated in patients ≥ 35 years of age[4]	<u>COPD</u><br>OHIP diagnostic codes: 491, 492, 496<br><br>ICD-10 diagnostic codes: J41, J42, J43, J44; ICD-9 diagnostic codes: 491, 492, 496	Ever
Organ transplantation: defined as history of transplantation or relevant follow-up (e.g., all transplant and organ procurement procedures)	CORR	Treatment codes: 171, 181	5 years prior
Hypertension	OHIP<br><br>CIHI-DAD, CIHI-SDS<br><br>An ICES-specific hypertension database was used to identify patients ever diagnosed with hypertension, based on ≥1 CIHI-DAD diagnoses or ≥2 OHIP diagnoses in a 2-	OHIP diagnostic codes: 401, 402, 403, 404, or 405<br><br>ICD-10 diagnostic codes: I10, I11, I12, I13, I15; ICD-9 diagnostic codes: 401, 402, 403, 404, 405	Ever

	year period; or 1 OHIP diagnosis followed by an OHIP/CIHI-DAD diagnosis within 2 years[6]		
Diabetes	OHIP<br><br>An ICES-specific diabetes database was used to identify patients ever diagnosed with diabetes, based on 3 OHIP diagnostic codes within 1 year[7]	OHIP diagnostic code: 250	Ever
Atrial fibrillation[8]	OHIP<br><br>NACRS-ED, CIHI-DAD	OHIP diagnostic codes: 427 (4 claims in 1 year, with max. 1 claim/day)<br><br>ICD-10 diagnostic code: I48 as any diagnosis type, including suspect.	5 years prior
Chronic kidney disease (CKD)	OHIP<br><br>CIHI-DAD, CIHI-SDS	OHIP diagnostic codes: 403, 585<br><br>ICD-10 diagnostic codes: E102, E112, E132, E142, I12, I13, N08, N18, N19	5 years prior
Human immunodeficiency virus (HIV)	OHIP<br><br>An ICES-specific HIV database was used to identify patients ever diagnosed with HIV, based on 3 OHIP diagnostic codes in 3 years[9]	OHIP diagnostic codes: 042, 043, 044	Ever
Cancer	Ontario Cancer Registry (OCR)	All except for non-melanoma skin cancer (ICD-10 O-3 Topography = C44 and Morphology = 87xx3)[10]	5 years prior
Frailty	CIHI-DAD, OHIP	Determined using the Johns Hopkins' ACG® System Version 10, using a set of	2 years prior to January 1st, 2020

		diagnosis codes that describe clinically frail individuals and are highly associated with marked functional limitations among older individuals.	
Smoking	Public Health Unit (PHU)-level regional rates of smoking were calculated by Public Health Ontario from national survey data. These rates were age-standardized and sex-specific.	Adapted from the Association of Public Health Epidemiologists in Ontario (APHEO) smoking status indicators. Rates and associated variances were calculated using methodology described on the Association of Public Health Epidemiologists in Ontario website [11]	2015 to 2016
Obesity	Public Health Unit (PHU)-level regional rates of obesity were calculated by Public Health Ontario from national survey data. These rates were age-standardized and sex-specific.	Adapted from the APHEO nutrition and healthy weights indicators. Rates and associated variances were calculated using methodology described on the Association of Public Health Epidemiologists in Ontario website [11]	2015 to 2016
Racial/ethnic diversity	Public Health Unit (PHU)-level regional rates of visible minority population were calculated by Public Health Ontario from national census data.	Per cent (%) visible minority population for both sexes, males, females (household measures only have one overall measure).	2016
Receipt of 2019-20 influenza vaccination<br><br>**N.B.** Persons receiving vaccinations outside of doctor's offices and pharmacies will not be captured by these codes. Otherwise, identifies individuals of all ages.	OHIP<br><br>ODB	OHIP billing codes: G590, G591, G592, Q130, Q590, Q690, Q691<br><br>ODB claim with any of the following Drug Identification Numbers (DINs): 02015986, 02223929, 02269562, 02346850, 02362384, 02365936, 02420643, 02420686, 02420783, 02426544, 02428881,	Between September 1st, 2019 and the index event

		02432730, 02473283, 09857501	
Receipt of COVID-19 vaccination (during end of second half of 2020 only)	COVaxON	Thorough, real-time documentation of all COVID-19 vaccination events in Ontario, including product, date of being administered, and dose number, is recorded into COVaxON, a centralized COVID-19 vaccine information system.	From the start of the wave until 2 weeks prior to the index date

APHEO = Association of Public Health Epidemiologists in Ontario, CCI=Canadian Classification of Health Interventions, CCP=Canadian Classification of Procedures, CORR=Canadian Organ Replacement Register, CHF=Ontario Congestive Heart Failure Database, DAD=Discharge Abstract Database, HIV=Ontario HIV database, ICD-9 = International Classification of Diseases, Ninth Revision, ICD-10 = International Classification of Diseases, Tenth Revision, NACRS=National Ambulatory Care Reporting System, OCR=Ontario Cancer Registry, ODB=Ontario Drug Benefit, ODD=Ontario Diabetes Database, OHIP=Ontario Health Insurance Plan, ORRS=Ontario Renal Reporting System, SDS=Same Day Surgery.

Supplemental Table 2. Baseline Characteristics of Community-Dwelling Individuals with and without SARS-CoV-2 Infection in Ontario, Canada between the weeks of June 14 to December 27, 2020

Characteristic		Negative Test (n=2,467,200)	Positive Test (n=122,290)	P-value
Week of testing (start date)	24 (June 14)	127011 (5.1%)	3340 (2.7%)	<0.001
	25 (June 21)	145860 (5.9%)	4651 (3.8%)	
	26 (June 28)	97247 (3.9%)	2350 (1.9%)	
	27 (July 5)	92999 (3.8%)	2008 (1.6%)	
	28 (July 12)	88920 (3.6%)	2041 (1.7%)	
	29 (July 19)	87355 (3.5%)	1909 (1.6%)	
	30 (July 26)	86382 (3.5%)	1715 (1.4%)	
	31 (Aug 2)	74866 (3.0%)	1371 (1.1%)	
	32 (Aug 9)	77089 (3.1%)	1433 (1.2%)	
	33 (Aug 16)	72562 (2.9%)	1379 (1.1%)	
	34 (Aug 23)	73487 (3.0%)	1624 (1.3%)	
	35 (Aug 30)	73236 (3.0%)	1648 (1.3%)	
	36 (Sep 6)	75874 (3.1%)	2160 (1.8%)	
	37 (Sep 13)	99552 (4.0%)	3119 (2.6%)	
	38 (Sep 20)	112903 (4.6%)	3858 (3.2%)	
	39 (Sep 27)	102495 (4.2%)	4035 (3.3%)	
	40 (Oct 4)	88302 (3.6%)	3508 (2.9%)	
	41 (Oct 11)	77471 (3.1%)	4119 (3.4%)	
	42 (Oct 18)	76116 (3.1%)	4769 (3.9%)	
	43 (Oct 25)	70231 (2.8%)	4592 (3.8%)	
	44 (Nov 1)	68496 (2.8%)	5420 (4.4%)	
	45 (Nov 8)	73317 (3.0%)	6668 (5.5%)	
	46 (Nov 15)	78868 (3.2%)	6710 (5.5%)	
	47 (Nov 22)	78286 (3.2%)	7133 (5.8%)	
	48 (Nov 29)	81205 (3.3%)	7762 (6.3%)	
	49 (Dec 6)	86278 (3.5%)	7956 (6.5%)	
	50 (Dec 13)	84847 (3.4%)	8456 (6.9%)	
	51 (Dec 20)	66454 (2.7%)	8122 (6.6%)	
	52 (Dec 27)	49491 (2.0%)	8434 (6.9%)	
**Sociodemographic factors**				
Age, years		45 (31-60)	42 (29-56)	<0.001
Age group, years	18-45	1,263,649 (51.2%)	67,721 (55.4%)	<0.001
	46-65	786,896 (31.9%)	40,497 (33.1%)	
	66-75	236,226 (9.6%)	7,993 (6.5%)	
	76-85	120,065 (4.9%)	3,801 (3.1%)	
	85+	60,364 (2.4%)	2,278 (1.9%)	
Sex	Female	1,389,467 (56.3%)	62,314 (51.0%)	<0.001
	Male	1,077,733 (43.7%)	59,976 (49.0%)	
Income quintile	1	457,970 (18.6%)	28,905 (23.6%)	<0.001
	2	474,667 (19.2%)	27,024 (22.1%)	
	3	493,123 (20.0%)	27,031 (22.1%)	
	4	504,787 (20.5%)	21,679 (17.7%)	
	5	536,653 (21.8%)	17,651 (14.4%)	
Community size	<10,000	247,173 (10.0%)	4,462 (3.6%)	<0.001
	10,000-100,000	192,643 (7.8%)	4,164 (3.4%)	
	100,000-500,000	533,259 (21.6%)	15,308 (12.5%)	
	500,000-1.5 million	421,664 (17.1%)	15,666 (12.8%)	
	≥1.5 million	1,072,461 (43.5%)	82,690 (67.6%)	
Rural dwelling		247,479 (10.0%)	4,455 (3.6%)	<0.001
Canadian immigrant		393,317 (15.9%)	46,006 (37.6%)	<0.001
Regional smoking rate, %		12 (9-16)	12 (9-13)	<0.001
Regional obesity rate, %		20 (15-24)	17 (15-22)	<0.001
Regional racial/ethnic diversity rate, %		26 (7-51)	51 (19-52)	<0.001
**Clinical risk factors**				
Hypertension		597,648 (24.2%)	28,304 (23.1%)	<0.001

Hypertension duration, years		13 (6-20)	11 (5-18)	<.001
Diabetes		231,874 (9.4%)	14,694 (12.0%)	<.001
Coronary artery disease		73,553 (3.0%)	2,761 (2.3%)	<.001
Heart failure		26,672 (1.1%)	851 (0.7%)	<.001
Stroke		12,699 (0.5%)	432 (0.4%)	0.429
Atrial fibrillation		71,457 (2.9%)	2,235 (1.8%)	<.001
Chronic kidney disease		73,364 (3.0%)	3,138 (2.6%)	<.001
HIV		5,422 (0.2%)	258 (0.2%)	0.521
Organ transplantation		3,667 (0.1%)	152 (0.1%)	0.03
Cancer		91,472 (3.7%)	2,237 (1.8%)	<.001
Liver disease		9,415 (0.4%)	245 (0.2%)	<.001
Lung disease		505,411 (20.5%)	21,161 (17.3%)	<.001
Hospitalization or ED visits in 2019	0	1,690,448 (68.5%)	87,347 (71.4%)	<.001
	1-2	625,161 (25.3%)	30,332 (24.8%)	
	3+	151,591 (6.1%)	4,611 (3.8%)	
Frailty		75,044 (3.0%)	2,586 (2.1%)	<.001
Influenza vaccination in 2019		656,438 (26.6%)	23,438 (19.2%)	<.001
COVID-19 vaccination	Part	*1-5	0 (0.0%)	0.753
	None	*2,467,195-2,467,199	122,290 (100.0%)	

Continuous variables are reported as median (interquartile range). Abbreviations: ED: emergency department; HIV: human immunodeficiency virus; SD: standardized difference. While in the early stages of modelling, there was significant heterogeneity between age and week of testing (P-interaction <0.0001), hence results are presented stratified prior to and following the peak of the first wave of the pandemic until the end of 2020.
*Not reported due to small cells.

Supplemental References

1. Vogel L. CMAJ News - COVID-19: A timeline of Canada's first-wave response. CMAJ. Published 2020. https://cmajnews.com/2020/06/12/coronavirus-1095847/
2. Nielsen K. A timeline of COVID-19 in Ontario. Global News. Published 2020. Accessed March 14, 2022. https://globalnews.ca/news/6859636/ontario-coronavirus-timeline/
3. Gershon AS, Wang C, Guan J, Vasilevska-Ristovska J, Cicutto L, To T. Identifying Patients with Physician-Diagnosed Asthma in Health Administrative Databases. *Can Respir J*. 2009;16(6):183-188. doi:10.1155/2009/963098
4. Gershon AS, Wang C, Guan J, Vasilevska-Ristovska J, Cicutto L, To T. Identifying Individuals with Physcian Diagnosed COPD in Health Administrative Databases. *COPD J Chronic Obstr Pulm Dis*. 2009;6(5):388-394. doi:10.1080/15412550903140865
5. Griffin MR, Zhu Y, Moore MR, Whitney CG, Grijalva CG. U.S. Hospitalizations for Pneumonia after a Decade of Pneumococcal Vaccination. *N Engl J Med*. 2013;369(2):155-163. doi:10.1056/NEJMoa1209165
6. Tu K, Campbell NRC, Chen Z-L, Cauch-Dudek KJ, McAlister FA. Accuracy of administrative databases in identifying patients with hypertension. *Open Med*. 2007;1(1):e18–e26. https://www.ncbi.nlm.nih.gov/pmc/articles/PMC2801913/
7. Lipscombe LL, Hwee J, Webster L, Shah BR, Booth GL, Tu K. Identifying diabetes cases from administrative data: a population-based validation study. *BMC Health Serv Res*. 2018;18(1):316. doi:10.1186/s12913-018-3148-0
8. Tu K, Nieuwlaat R, Cheng SY, et al. Identifying Patients With Atrial Fibrillation in Administrative Data. *Can J Cardiol*. 2016;32(12):1561-1565. doi:10.1016/j.cjca.2016.06.006
9. Antoniou T, Zagorski B, Loutfy MR, Strike C, Glazier RH. Validation of Case-Finding Algorithms Derived from Administrative Data for Identifying Adults Living with Human Immunodeficiency Virus Infection. Thiem U, ed. *PLoS One*. 2011;6(6):e21748. doi:10.1371/journal.pone.0021748
10. Hall S, Schulze K, Groome P, Mackillop W, Holowaty E. Using cancer registry data for survival studies: the example of the Ontario Cancer Registry. *J Clin Epidemiol*. 2006;59(1):67-76. doi:10.1016/j.jclinepi.2005.05.001
11. Association of Public Health Epidemiologists in Ontario. Methods for Age Standardizing Survey Estimates. Published 2018. https://www.apheo.ca/methods-for-age-standardizing-survey-estimates
